# Gallant's Corsets in the Treatment of Enteroptosis

**Published:** 1906-05-12

**Authors:** 


### GALLANT'S CORSETS IN THE TREATMENT OF ENTEROPTOSIS.

In a lecture on Glénard's disease, hepatoptosis, and movable kidney Mr. Watson Cheyne[1] draws attention to the value of the corsets devised by Gallant. In the use of these it is necessary in the first place to allow the prolapsed viscera to resume their natural position by gravity. Hence before their application the patient is placed in the recumbent posture, and the return of the organs is facilitated by rubbing or stroking the abdomen. While in this position the patient is measured for a corset, which must fit closely over the hips and suprapubic region, but must afford sufficient room above the umbilicus for the replaced colon and stomach. In the third place, when putting on the corset the patient places it around her waist, lies down upon the bed or couch, flexes her knees, and raises her hips so as to further the visceral replacement, and, while in this posture, fastens the corset, beginning from below upwards. Fourthly, in lacing the corset two strings are used, one beginning at the bottom and lacing up to the first eyelet below the waist line, and this lace must be drawn as tightly as it can be borne; the second begins where the lower leaves off, is laced up to the top, and must never be drawn tight. Any tendency of the corset to ride upwards indicates that it has not been laced tightly enough below the waist, or that it has been laced too tightly above, or that the corset is too large. It is claimed that with this corset belts, binders, and pads may be done away with, though where the patient is spare a pad may be placed in each iliac fossa. In addition to enteroptosis the corsets are of value in the treatment of movable kidney.

[1] Lancet, April 7, 1906.

